# Supplementary material for: Neutrophil prime unique transcriptional responses in intestinal organoids during infection with nontyphoidal Salmonella enterica serovars
Source: mSphere. 2024 Nov 20;9(12):e00693-24. doi: 10.1128/msphere.00693-24 (PMC11656734; doi:10.1128/msphere.00693-24)
Supplement: Supplemental Figures — Fig. S1 to S5. [file msphere.00693-24-s0001.pdf]

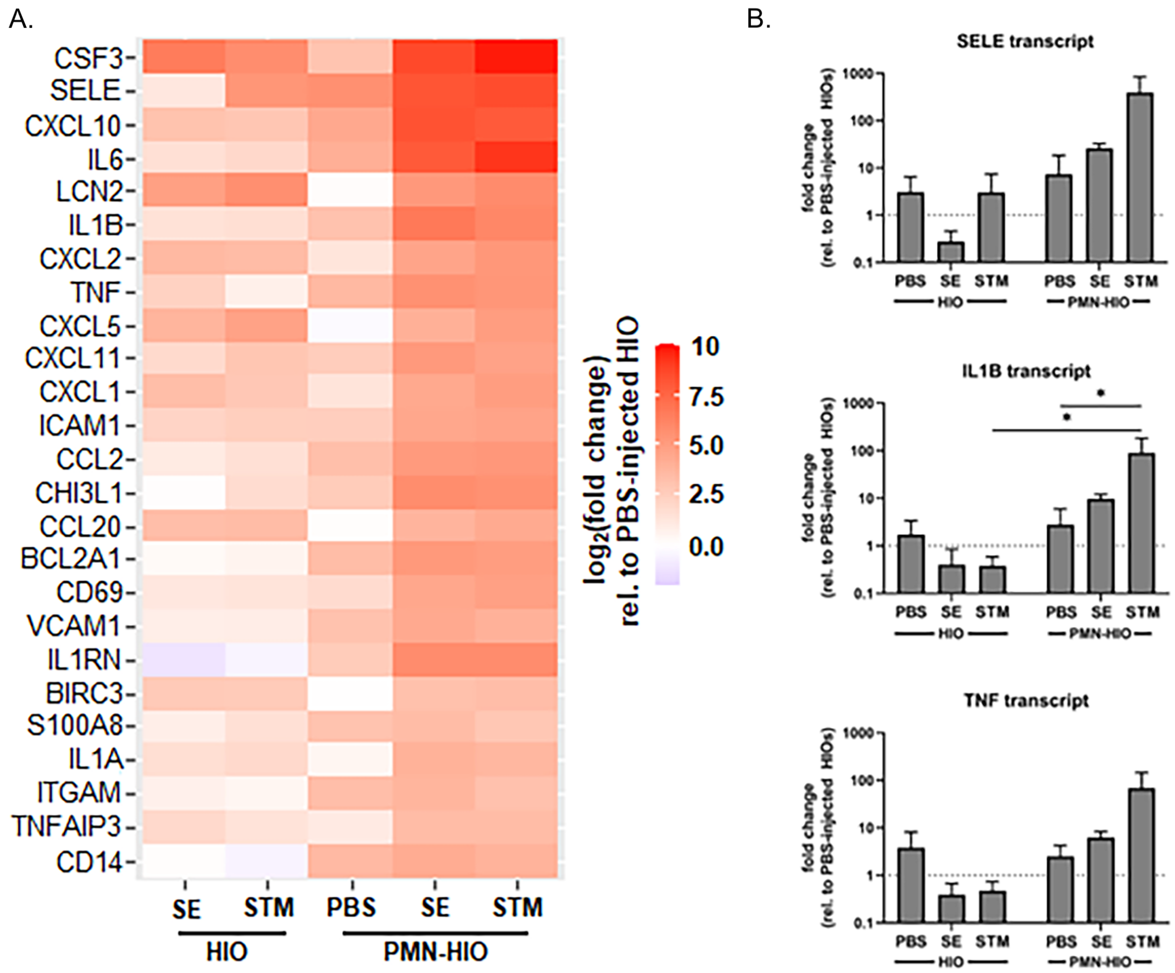

FIG S1 Heatmap of top 25 diarrhea associated genes and qRT-PCR validation of diarrhea associated genes

(A) Top 25 diarrhea associated genes presented as log_2(fold change) relative to PBS-injected HIOs. (B) Quantitative real-time PCR (qRT-PCR) validation of diarrhea associated genes in HIOs and PMN-HIOs at 8hpi. Results represent data from n=4 replicates and significance was determined by 2-way ANOVA where $*p<0.05$.

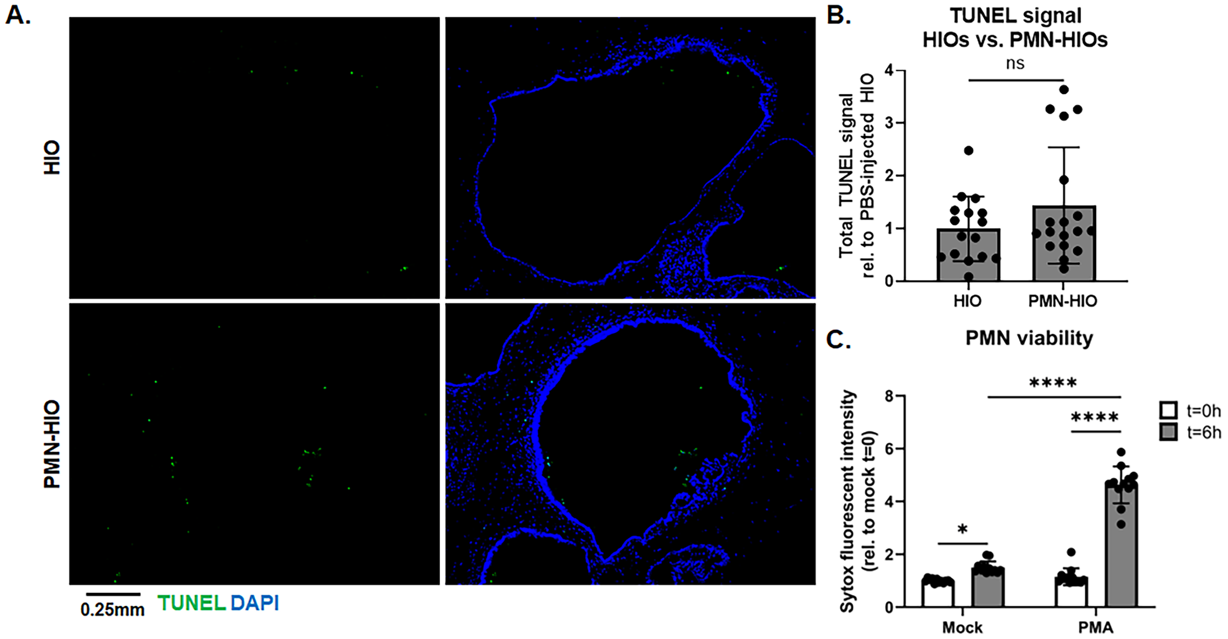

FIG S2 Presence of PMNs does not significantly increase cell death in HIOs

(A) TUNEL staining of PBS-injected HIOs and PMN-HIOs at 8h of co-culture. Total TUNEL signal was quantified and normalized to total DAPI signal. Results represent data from n=2 independent experiments with at least 16 HIOs quantified. TUNEL (green) DAPI (blue). (B) Quantitation of (A). Significance was determined by unpaired t-test. (C) Sytox assay quantifying cell death in PMNs relative to freshly isolated PMNs. PMNs were tested 6h after culture in ENR media which corresponds to the same duration as in the co-culture experiments. 100nM PMA was used as a positive control to induce PMN cell death. Technical replicates are plotted from a representative experiment from n=3 separate experiments with different donors. Significance was determined by 2-way ANOVA where $*p<0.05$, $****p<0.0001$.

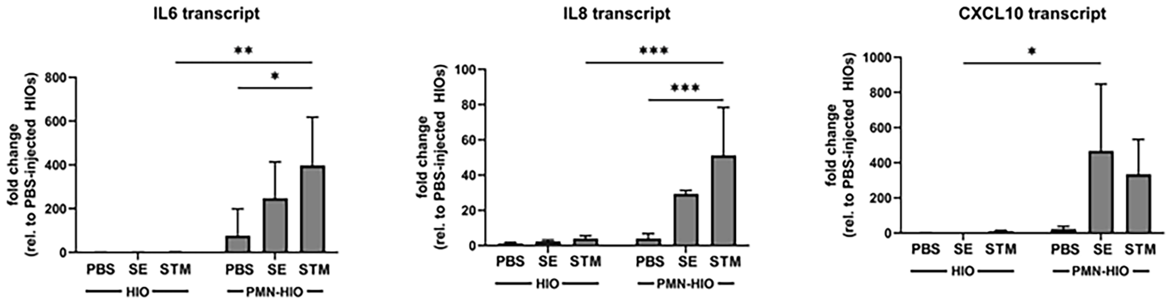

FIG S3 qRT-PCR validation of cytokine and chemokine genes

qRT-PCR validation of select cytokine and chemokine genes in HIOs and PMN-HIOs at 8hpi. Results represent data from n=4 replicates and significance was determined by 2-way ANOVA where *p<0.05, **p<0.01, ***p<0.001.

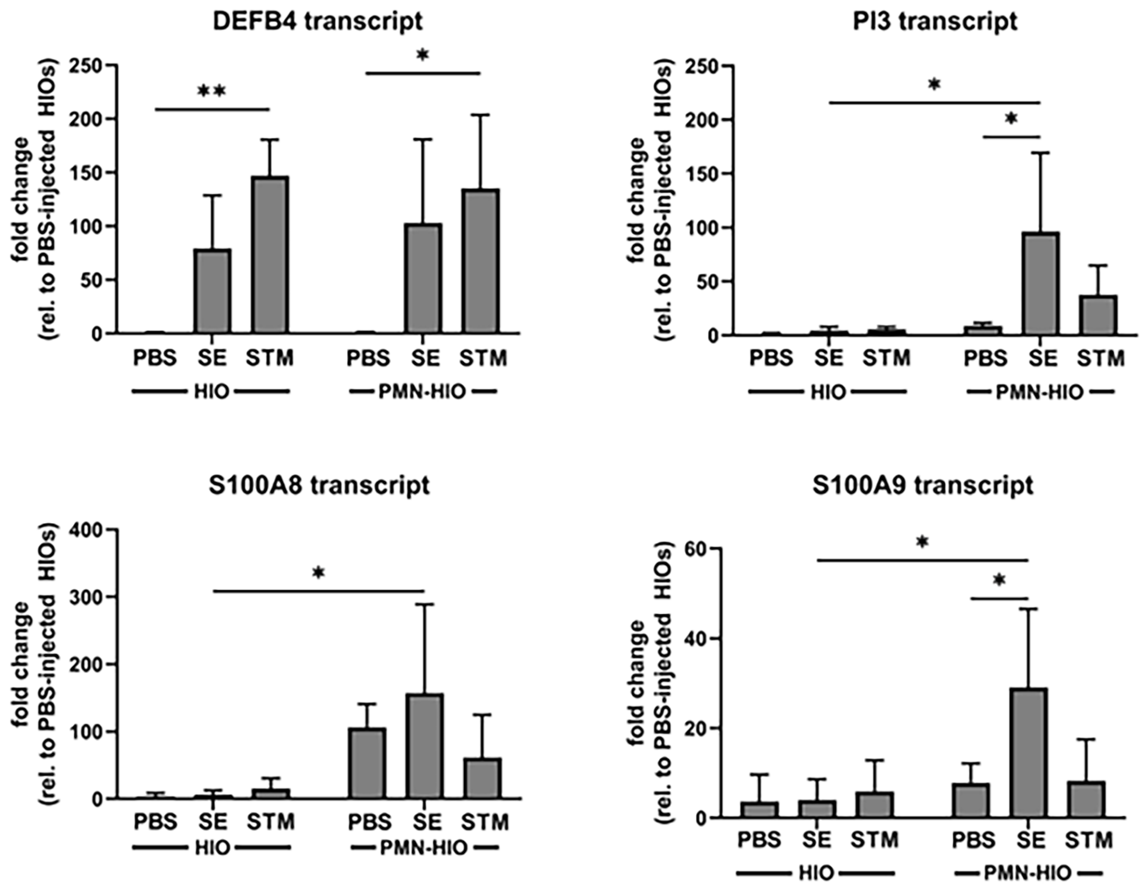

FIG S4 qRT-PCR validation of antimicrobial genes

qRT-PCR validation of select antimicrobial genes in HIOs and PMN-HIOs at 8hpi. Results represent data from n=4 replicates and significance was determined by 2-way ANOVA where *p<0.05, **p<0.01.

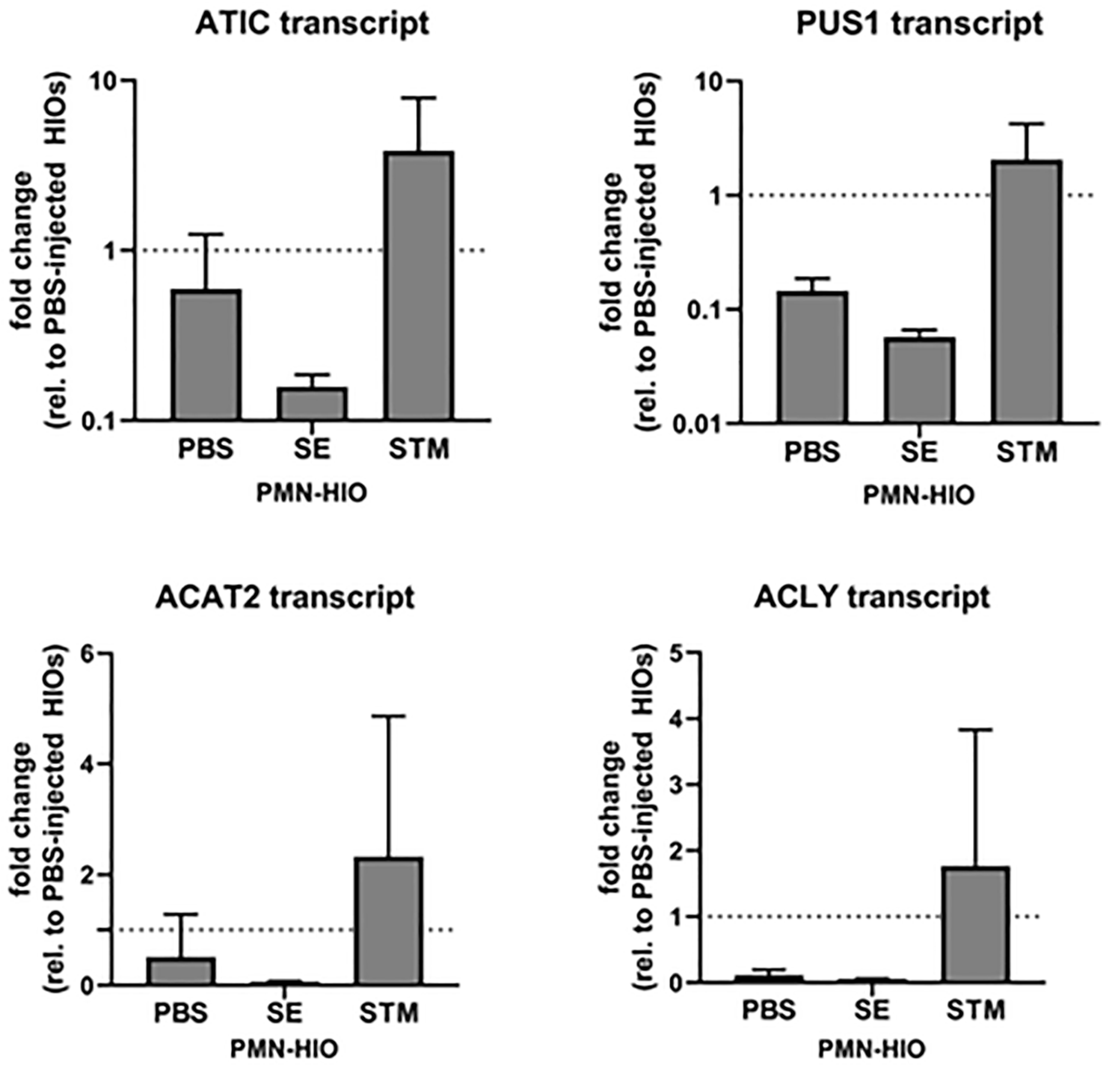

FIG S5 qRT-PCR validation of serovar-specific genes

qRT-PCR validation of select genes that were found to be uniquely regulated by STM or SE in PMN-HIOs at 8hpi. Results represent data from n=4 replicates and significance was determined by 2-way ANOVA.
